# The DarT/DarG Toxin–Antitoxin ADP-Ribosylation System as a Novel Target for a Rational Design of Innovative Antimicrobial Strategies

**DOI:** 10.3390/pathogens12020240

**Published:** 2023-02-02

**Authors:** Giuliana Catara, Rocco Caggiano, Luca Palazzo

**Affiliations:** 1 Institute of Biochemistry and Cell Biology, National Research Council of Italy, CNR, 80131 Naples, Italy; 2 Institute for the Experimental Endocrinology and Oncology, National Research Council of Italy, CNR, 80131 Naples, Italy; 3 Department of Molecular Medicine and Medical Biotechnology, University of Naples “Federico II”, 80131 Naples, Italy

**Keywords:** ADP-ribosylation, toxin–antitoxin, DarT/DarG, DNA modification, cell growth, phage defence, antimicrobial resistance

## Abstract

The chemical modification of cellular macromolecules by the transfer of ADP-ribose unit(s), known as ADP-ribosylation, is an ancient homeostatic and stress response control system. Highly conserved across the evolution, ADP-ribosyltransferases and ADP-ribosylhydrolases control ADP-ribosylation signalling and cellular responses. In addition to proteins, both prokaryotic and eukaryotic transferases can covalently link ADP-ribosylation to different conformations of nucleic acids, thus highlighting the evolutionary conservation of archaic stress response mechanisms. Here, we report several structural and functional aspects of DNA ADP-ribosylation modification controlled by the prototype DarT and DarG pair, which show ADP-ribosyltransferase and hydrolase activity, respectively. DarT/DarG is a toxin–antitoxin system conserved in many bacterial pathogens, for example in *Mycobacterium tuberculosis*, which regulates two clinically important processes for human health, namely, growth control and the anti-phage response. The chemical modulation of the DarT/DarG system by selective inhibitors may thus represent an exciting strategy to tackle resistance to current antimicrobial therapies.

## 1. Introduction

ADP-ribosylation is a reversible post-translational modification (PTM) found in all three domains of life, as well as in several viruses [1,2,3,4]. It was identified in the 1970s as a key enzymatic activity required for cholera and diphtheria toxin pathogenesis [5,6]. Since then, the understanding of ADP-ribosylation has increased, from bacteria to mammals. Today, it is mainly known in the scientific community for its key role in DNA damage repair [7,8,9,10] and for being the target of tailored cancer therapies [11,12,13,14]. However, the functions of ADP-ribosylation are also vital for controlling many additional physiological processes, such as transcription and translation [15,16,17,18,19,20,21], cell proliferation [22,23], and cell death [24,25,26] along with stress and immune responses [27,28,29,30,31,32,33] and many others [34,35,36]. The control of cell homeostasis in both prokaryotes and eukaryotes by ADP-ribosylation [19,34,35,37,38,39,40,41] makes this a PTM of great interest in many fields of human health. 

ADP-ribosylation is characterised by the addition of ADP-ribose unit(s) from nicotinamide adenine dinucleotide (NAD^+^) onto cellular substrates with the release of nicotinamide [42,43]. Consistent with the number of ADP-ribose moieties attached, single or multiple, the reaction is further differentiated into mono-ADP-ribosylation (MARylation) and poly-ADP-ribosylation (PARylation), respectively [44]. It was originally discovered as a PTM mainly targeting proteins [45], but it can also be covalently attached to additional macromolecules, including DNA [38,46,47,48,49] and RNA [48,50], as well as to small molecules such as antibiotics [51], ATP, and ADP [36]. 

There is a balanced interplay between specialised enzymes, namely, ADP-ribosyltransferases and ADP-ribosylhydrolases, which are responsible for the attachment and removal of the modification from cellular targets, respectively. This interplay shapes ADP-ribosylation signalling [43,44,52,53,54]. The dysregulation of these enzymatic activities in humans thus has implications in the pathogenicity of several diseases, above all, neurological disorders [55,56], cancer [57,58], and bacterial- and viral-mediated infections, as discussed here [59,60,61,62]. 

ADP-ribosylation is currently known to be involved in a large number of infectious diseases worldwide [63,64,65,66], including COVID-19 [33,60,61,67,68,69,70,71,72], Legionnaires’ disease [73,74,75,76,77], and the infectious diseases caused by the virulent *M. tuberculosis*. From a pathogenic point of view, the mechanisms of ADP-ribosylation in *M. tuberculosis* infection involve the modification of their own/endogenous targets rather than the host proteins, ultimately enabling the cell to adapt within the host and to improve the biological fitness. Among these mechanisms, the activity of the DarT/DarG toxin–antitoxin (TA) ADP-ribosylation system in *M. tuberculosis* targets bacterial genomic DNA. As a result of DNA modification, ADP-ribosylation slows growth and potentially induces bacterial persistence, a phenotypic state that correlates with antibiotic resistance [38,40,78]. 

The excessive use of antibiotics to counteract pathogen infections has led to the spread of antibiotic resistant “superbugs”, which currently represent a major public health threat [79,80]. Antibiotic resistance occurs in a wide range of bacterial infections and is determined by the activation of pathogen resistance/defence mechanisms, which also enable the cells to become persistent and tolerant to antibiotics [81,82]. Bacterial TA systems are widespread in Gram-negative and Gram-positive bacteria, and are involved in cell regulatory mechanisms in response to stress stimuli [83,84,85], including antibiotic resistance. Targeting TA modules such as the ADP-ribosylation DarT/DarG system can thus act as a blueprint for designing alternative drugs to current therapeutic treatments of antibiotic-resistant pathogens [86,87].

In this perspective, we discuss the structural and mechanistic aspects of DarT/DarG toxin–antitoxin-mediated control of DNA ADP-ribosylation. In addition, we then address the pathogenic role of the DarT/DarG TA pair as well as the therapeutic perspectives that the modulation of this specific ADP-ribosylation signalling may have.

## 2. ADP-Ribosylation in Bacteria 

### 2.1. NAD^+^-Dependent Endotoxins and Exotoxins Involved in ADP-Ribosylation Signalling

**Figure 1:**
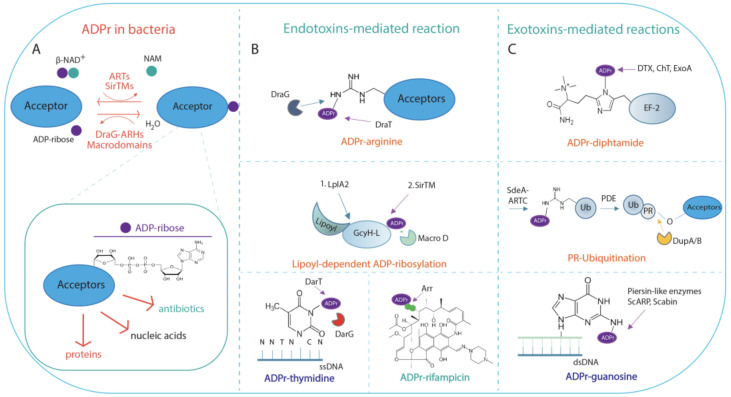
Schematic representation of ADP-ribosylation reaction in bacterial meta-cell. (**A**) ADP-ribosylation (ADPr) reaction is catalysed by NAD^+^-dependent diphtheria toxin-like ARTs ARTDs, the cholera toxin-like ARTs ARTCs, or SirTMs, which transfer a single ADP-ribose unit on acceptors. Macrodomain-containing hydrolases (Macrodomains) or DraG-related ADP-ribosylhydrolases reverse the reaction by generating free ADP-ribose and unmodified acceptor. Inset: ADP-ribose moiety linked to acceptor substrates, which can be proteins, nucleic acids, or antibiotics. (**B**) Endotoxin-mediated reaction. Endotoxins can modify proteins, nucleic acids, or antibiotics. ARTDs, ARTCs, and SirTM modify endogenous bacterial substrates on different residues as indicated. ADP-ribosylated (ADPr)-arginine: MARylation of arginine residue is performed by the DraT enzyme, and is reversed by the cognate DraG ADP-ribosylhydrolase. Further examples are provided in the text. Lipoyl-dependent MARylation is carried out by SirTM and is dependent on prior lipoylation of the lipoyl-carrier protein GcyH-L, by the lipoate-protein ligase A (LplA2). The modification is reversed by the MacroD hydrolase, which is encoded within the same SirTM operon; ADP-ribosylated (ADPr)-thymidine: the reaction is performed by the endotoxin DarT that modifies thymidine base on ssDNA; the cognate DarG antitoxin reverses the modification; ADP-ribosylated (ADPr)-rifampicin: MARylation of the rifampin antibiotic is catalysed by Arr toxin. (**C**) Exotoxin-mediated reactions. ARTDs and ARTCs modify host targets on different residues as indicated. ADP-ribosylated (ADPr)-diphthamide: the reaction is catalysed by the toxins DTX, ChT, and ExoA, which irreversibly transfer ADP-ribose on the residue diphthamide on the elongation factor 2; PR-Ubiquitination. SdeA toxin catalyses the ADP-ribosylation (ADPr)-dependent ubiquitination of host proteins in a two-step reaction as detailed in the text. The reaction is reversed by the phosphodiesterases DubA/B; ADP-ribosylated (ADPr)-guanosine. The irreversible ADP-ribosylation on guanosine in dsDNA is performed by the pierisin-like enzymes ScARP and Scabin.

ADP-ribosylation sustains prokaryotic cells in both cell metabolic processes and pathogenic mechanisms through the activity of NAD^+^-dependent enzymes, namely, endotoxins and exotoxins, respectively (Figure 1A–C). 

The majority of endotoxins and exotoxins belong to the superfamily of ADP-ribosyltransferase (ART) enzymes, which, despite limited sequence similarity, share a conserved secondary structure and protein fold [2,88]. ARTs fall into three phylogenetically distinct clades according to the catalytic triad composition: (*i*) the diphtheria toxin-like ARTs (ARTDs), characterised by the catalytic H-Y-[EDQ] triad; (*ii*) the cholera toxin-like ARTs (ARTCs), harbouring the R-S-E motif in the catalytic domain; and (*iii*) the tRNA 2’-phosphotransferase (TpT1/KptA) containing the H-H-h motif, with h containing any hydrophobic residues [2,43,52]. In addition to these subgroups, in *Staphylococcus aureus* and *Streptococcus pyogenes*, the microbial SirTMs, which belong to the Sirtuin superfamily of enzymes, catalyse the lipoyl-dependent ADP-ribosylation of proteins following a non-canonical deacylation reaction [89]. 

The substrate selectivity of ARTs is provided by two conserved functional motifs called the ARTT loop (ADP-ribosylating turn-turn), which is also known as the acceptor-loop (A-loop), and the donor-loop (D-loop). The D-loop is exclusive to ARTDs. Both loop structures are evolutionarily highly conserved, although their amino acid sequence and length vary greatly among the ARTDs [43]. In comparison with eukaryotic ARTs, bacterial enzymes show narrow amino acid residues specificity in host targets. Bacterial ARTs are in fact able to MARylate target proteins on several amino acid residues (i.e., arginine, cysteine, threonine, asparagine, and glutamine for ARTCs, diphthamide for ARTDs) (Figure 1B,C). Unlike some mammalian ART homologues (namely, PARP1 and PARP2) [90,91,92], bacterial ARTs do not need specificity factors [43]. In addition, bacterial ADP-ribosyltransferases can also modify nucleic acids (Figure 1B,C) [38,93]. Some bacterial ARTs, such as the ARTD homologue Arr-ms from *Mycobacterium smegmatis,* can also ADP-ribosylate the hydroxyl group at C23 of rifamycin and derivatives, thus inactivating antibiotics [51,94] (Figure 1B).

ADP-ribosylation is a reversible PTM. Two structurally distinct families of ADP-ribosylhydrolases, namely, DraG-related ADP-ribosylhydrolases (ARHs) and macrodomain-containing ADP-ribosylhydrolases, revert ADP-ribosylation signalling in bacterial cells (Figure 1A) [4]. 

DraG-related ARHs, from the founder DraG protein found in the nitrogen-fixing bacterial species *R. rubrum* and *A. brasilense* [37,95], are compact and globular modules with a typical domain length of 290–360 residues, with a central core motif comprising 13 orthogonal α-helices and a variable number of supplementary helices depending on the organism and type. The divalent metal ions enable the correct positioning of the substrate in the active site [53]. Structural studies on *R. rubrum* DraG hydrolase show that the de-MARylation of substrates occurs via the opening of the ribose ring and the formation of a protonated Schiff base. This substrate opening leads to a shift in metal coordination, allowing a nucleophilic attack by a water molecule activated by Mn^2+^ and resulting in a tetrahedral intermediate. The proton transfer via D97 promotes intermediate collapse and the release of arginine [96]. 

Macrodomain-containing hydrolases, harbouring the ADP-ribose-binding domain known as the macrodomain, share a conserved α/β/α fold consisting of a six-stranded mixed β-sheet surrounded by five α-helices [53]. Substrate binding takes place in a deep cleft on the top of the domain and several conserved interactions contribute to stabilise the ligand–macrodomain complex [53,54]. Based on ADP-ribosylhydrolase activity, macrodomains are further classified into mono-ADP-ribosylhydrolases (including MacroD-type and ALC1-like enzymes) and poly-ADP-ribosylhydrolases (PARG). Several bacterial macrodomains have been characterised as belonging to MacroD-, ALC1-, and PARG-like phylogenetically distinct groups that regulate a variety of cellular processes by deacetylating O-acetyl-ADP-ribose, and by hydrolysing MARylated targets, which include proteins and RNA [50,97,98]. In addition, several enzymes such as the TARG1-type macrodomain enzyme from *Fusobacterium mortiferum* ATCC 9817 [99] and the bacterial PARG from *Thermomonospora curvata* [97] have been reported to hydrolyse chains of ADP-ribose in vitro. The finding of an endogenous bacterial PARG-processing enzyme in *Deinococcus radiodurans* would seem to indicate an active prokaryotic PARylation machinery that may be involved in the stress response, given that PARG disruption leads to PAR enrichment in treated cells and loss of recovery after UV irradiation [100].

In a similar way to what happened for cancer treatment with the discovery of specific inhibitors of PARP1/PARP2 and PARG, the in-depth understanding of the enzymatic reactions and structural features of both bacterial ARTs and hydrolases promises important advances in antimicrobial therapies, which may eventually help to tackle antibiotic resistance.

### 2.2. Functional Aspects of ADP-Ribosylation in Bacteria 

From a functional point of view, endotoxins modify endogenous targets, thus regulating the oxidative stress response [89], morphological differentiation and antibiotic production [101,102], and the maintenance of cell homeostasis in response to environmental stimuli, as exemplified by the *Rhodospirillum rubrum* and *Azospirillum brasilense* DraT/DraG system that regulates nitrogen fixation [37,95,96,103]. On the other hand, exotoxins promote pathogenic mechanisms through the transfer of ADP-ribose onto host targets, which alters signal transduction (e.g., CTX from *Vibrio. cholerae*; ETEC from *Escherichia coli*), cellular cytoskeleton organisation along with membrane trafficking (e.g., C2 toxin from *Clostridium botulinum*; ExoS toxin from *Pseudomonas. aeruginosa*), and protein synthesis (e.g., DTX from *Corynebacterium diphtheriae;* ChT from *Vibrio cholerae*) [4,45,62]. Bacterial exotoxins appear to be involved in the aetiology of important diseases [4,62,104]. Of these, SidE family effectors regulate the pathogenicity of *Legionella pneumophila* by non-canonical phosphoribosyl ubiquitination (Figure 1C), which interferes with the host physiological ubiquitination machinery [73,76,105,106], ultimately leading to the impairment of mitophagy and the secretory pathway [107]. The SdeA toxin, which is one of SidE family effectors released by the pathogenic *L. pneumophila*, catalyses the ADP-ribosylation-dependent ubiquitination of host proteins in a two-step reaction. Firstly, SdeA transfers the ADP-ribose on arginine 42 of a ubiquitin (Ub) molecule to generate an ADP-ribosylated-Ub intermediate due to the presence of an ARTC domain; the ADP-ribosylated-Ub intermediate is then converted to phosphoribosyl-Ub by the SdeA phosphodiesterase activity and is then conjugated through an ester linkage to a serine residue to target protein. Cognate phosphodiesterases DubA/B revert the reaction (Figure 1C).

Of particular interest from this perspective is that recent discoveries have established nucleic acids, such as genomic DNA and RNAs, as novel ADP-ribosylation targets [48,49,50], which, although involved in crucial physiological processes, are not yet fully understood in either mammals or prokaryotes [47,48,50,108]. To date, the ADP-ribosylation of DNA has only been characterised in a few bacterial systems including pierisin-like members and the DarT/DarG system. Pierisin and the pierisin-like ARTs represent a small group of ARTC toxins that prevalently ADP-ribosylate DNA. Pierisin, which is the founder of the family, has been identified in the cabbage butterfly species, *Pieris rapae*, where it counteracts the non-habitual parasitoids [109]. Extracts from *P. rapae* are highly cytotoxic toward insect and mammalian cells. In fact, pierisin induces irreversible host apoptosis by ADP-ribosylating N2 amino groups of 2′-deoxyguanosine into double-stranded DNA (dsDNA) in a non-conserved sequence manner, and as such, pierisin likely plays a role in the defence mechanism [110]. Similarly, the bacterial pierisin-like Scabin from *Streptomyces scabies* [111] and ScARP from *Streptomyces coelicolor* [112] are able to modify DNA on the exocyclic amino group on guanine bases and most of its derivatives in either single-stranded (ssDNA) or dsDNA. The disruption of ScARP affects *S. coelicolor* morphological differentiation, sporulation, and increased antibiotic production [101]. 

Unlike pierisin-like ARTCs, the DarT toxin from *Thermus aquaticus* and *M. tuberculosis* can specifically modify genomic ssDNA on thymidine in the conserved nucleotide sequence NNTNTCN, which can strongly hinder bacterial cell growth and, in turn, can have implications in antimicrobial susceptibility. Importantly, the cellular effects on the bacterial growth induced by DarT can be neutralised by DarG hydrolase, which, by reverting DNA-ADP-ribosylation, acts as an antitoxin [38]. Section 3 details the DarT/DarG system. 

## 3. The DarT/DarG ADP-Ribosylation-Dependent System 

### 3.1. DarT Is a New PARP-Like Toxin and a Potential Molecular Target for Antimicrobial Therapy

**Figure 2:**
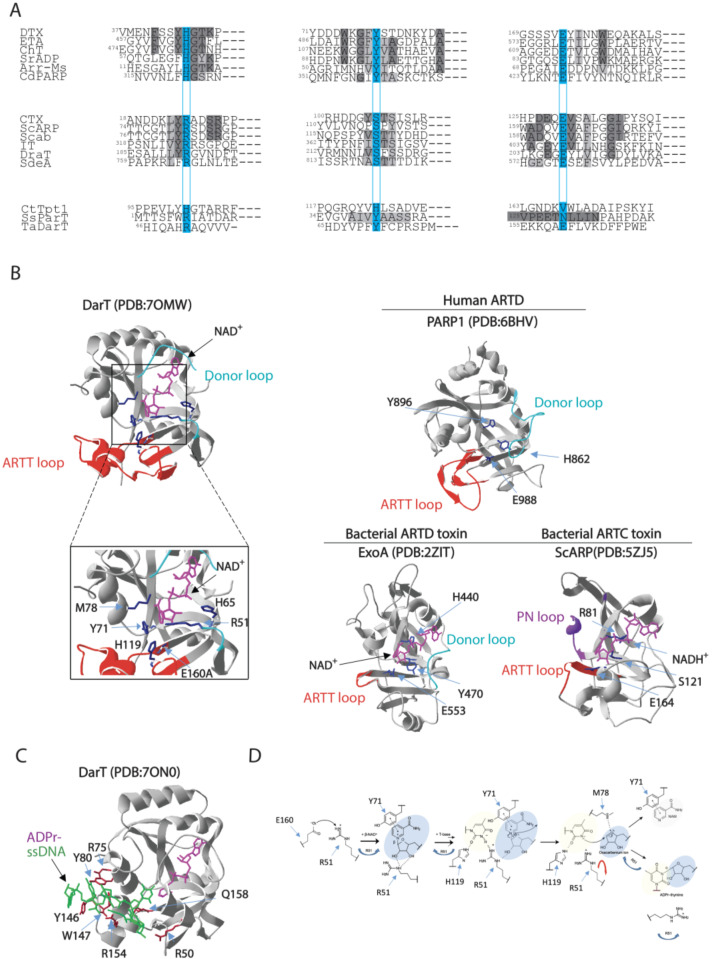
Comparison of amino acid sequences and 3D structures of representative ARTs. (**A**) Alignment of the partial sequences of the bacterial ARTs. ARTD members, which harbour the H-Y-E catalytic residues, include: DTX, diphtheria toxin from *C. diphtheriae;* ETA, exotoxin A from *P. aeruginosa*; Ch toxin, cholix toxin from *V. cholerae*; SrADP, toxin from *Streptomyces roseifaciens*; Arr-Ms, rifamycin ADP-ribosylation toxin from *Mycobacterium smegmatis*; Cd-PARP, toxin from *Clostridium perfringens* CD 160. ARTC members, which enclose R-S-E catalytic residues, include: CTX, cholera toxin from *V. cholerae*; ScARP, toxin from *S. coelicolor*; Scabin from *S. scabies*; IT, iota toxin from *C. perfringens*; DraT, dinitrogenase reductase ADP-ribosyltransferase from *R. rubrum;* SdeA, ADP-ribosylation-dependent ubiquitination toxin from *L. pneumophila*. Divergent enzymes include: CtTpt1, Tpt1 RNA-phosphotransferase enzyme from *Clostridium thermocellum*; ParT, ADP-ribosylating toxin of ParT/ParS TA system from *Sphingobium* sp. YBL2; TaDarT, DNA ADP-ribosylating toxin of DarT/DarG TA system from *T. aquaticus*. The residues involved in catalysis are boxed on a light blue background. Identities or accepted amino acid substitutions are indicated in dark and light grey, respectively. (**B**) Cartoon–stick model of *Thermus* sp. 2.9 DarT(E160A) (PDB:7OMW, [78]) showing the NAD^+^ binding site in complex with NAD^+^, the amino acid residues involved in the catalytic activity (blue), the regulatory ARTT-loop (red) and the donor-loop (light blue). Inset: the catalytic site residues R51, H65, Y71, M78, H119, and E160A localise in proximity of nicotinamide in the active site. Cartoon–stick models of the 3D structure of the human ARTD PARP1 (PDB:6BHV, [113]), the bacterial ARTD-toxin ExoA (PDB:2ZIT, [114]), and the bacterial ARTC-toxin ScARP (PDB:5ZJ5, [115]) are shown as exemplars. (**C**) Cartoon–stick model of *Thermus* sp. 2.9 DarT(E160A) (PDB:7ON0, [78]) in complex with ADP-ribosylated ssDNA showing the residues (dark red) involved in the interaction with ssDNA (green). (**D**) Catalytic mechanism proposed for DarT-mediated ADP-ribosylation reaction of DNA.

ADP-ribosylation catalysed by DarT specifically targets the thymidine bases present in conserved ssDNA sequence NNTNTCN in *T. aquaticus* and TTTT/A in *M. tuberculosis,* respectively, thereby showing no activity on dsDNA and RNA or protein targets. Substrate specificity toward a thymidine base also takes place for DarT toxin homologues, as highlighted in enteropathogenic *E. coli* DarT, which shows a preference for TCT/TTT sequences by modifying the third thymidine base of these motifs [40]. From a structural point of view, DarT can be considered a divergent ART enzyme given that it lacks the canonical catalytic triad residues found in ARTD and ARTC bacterial endotoxins (Figure 2A). Compared to bacterial ARTDs and ARTCs, DarT is very variable in terms of primary structure and motifs in comparison with bacterial ARTDs and ARTCs (Figure 2A). Nevertheless, the overall ART fold is maintained, as revealed by 3D resolution structural insights into *Thermus* sp. 2.9 DarT [78]. In fact, DarT is a PARP-like enzyme [78], as also predicted by phylogenetically analyses, as it is closer to human ARTDs than bacterial ART counterparts (Figure 2B). 

Secondary structure elements are found conserved such as the fold of the central six-stranded b-sheet core and the helices between strands β1 and β2 (β1–2) and β2 and β3 (β2–3). Target DNA binds to a solvent-accessible channel placed orthogonally to the NAD^+^ molecule (Figure 2B,C) and is stabilised by the ARTT loop, which is known to affect substrate specificity in ARTDs as mentioned before. The length of the ARTT loop in DarT exceeds the ARTT loop of bacterial ARTDs and is instead comparable to loops found in human ARTDs, including PARP1, PARP2, and PARP3, thus forming a stable scaffold for the DNA target. 

**Figure 3:**
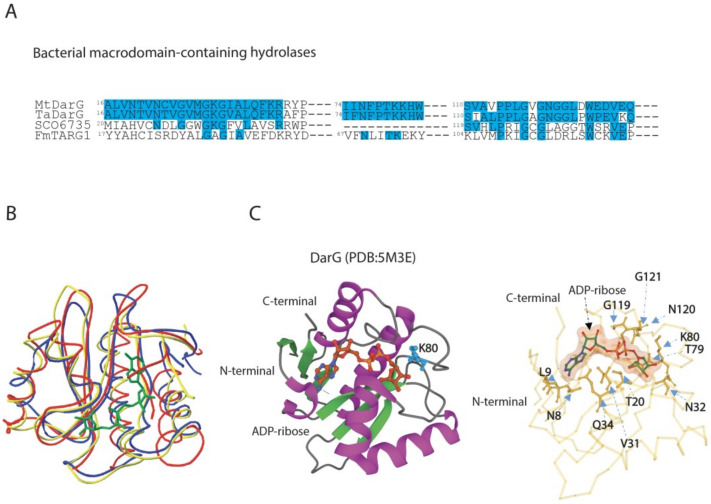
Comparison of amino acid sequences and 3D structures of macrodomain-containing hydrolases belonging to ALC1-like group. (**A**) Alignment of partial sequences of ALC1-like hydrolases from bacteria. MtDarG, DarG from *M. tuberculosis;* TaDarG, DarG from *T. aquaticus*; SCO6735, macrodomain-containing hydrolase from *S. coelicolor*; FmTARG1, TARG1 homologue from *F. mortiferum*. Identities are indicated in light blue. (**B**) Structural comparison between DarG from *T. aquaticus* in complex with ADP-ribose (yellow line, PDB: 5M3E, [38]), human TARG1 in complex with ADP-ribose (blue line, PDB:4J5S, [55]) and SCO6735 (red line, PDB:5E3B, [119]). (**C**) DarG from *T. aquaticus* (cartoon) in complex with ADP-ribose (ball and stick). The catalytic K80 is shown in light blue (left panel). Close up of the *T. aquaticus* DarG active site showing the residues involved in ADP-ribose binding (right panel).

Given that the ARTT loop is found conserved in human ARTDs and that several ARTDs also ADP-ribosylate DNA [47,116,117,118], it is tempting to speculate that the ARTT loop plays a role in the interaction with the DNA target. The DNA binding site is located in a groove enriched with basic amino acid residues that enable the formation of a series of interactions that serve the sequence-specific ADP-ribosylation by DarT, with thymidine targeted for ADP-ribosylation pointing orthogonally to the DNA backbone deep inside the active site of DarT. Additional interactions between the DNA fragment and DarT side chains and main chains, in addition to structural waters, stabilise the phosphate–ribose backbone. DarT does not exhibit any NADase activity or auto-ADP-ribosylation activity and shows a distinct catalytic mechanism in comparison with other ARTDs. DarT binds the NAD^+^ substrate within a large pocket with key interactions resulting in a binding mode of constrained conformation. The adenine moiety is stabilised by hydrogen bonding to the K28 and L30 backbone amides, the adenine–proximal ribose bonds with its 2′ and 3′ hydroxyl groups to T15/H13 and N19, respectively. On the other hand, the NAM moiety is permanently maintained in position by π–π interaction with Y71 and hydrogen bonding of its primary amide to I14 and intra-molecularly to the beta-phosphate [78]. DarT shows a diverse arrangement of the catalytic site, wherein a key role is played by R51 residue, which expands the canonical ART catalytic motifs’ repertoire. ADP-ribosylation reaction occurs in several steps including: (1) locking of the thymidine base in plane for ADP-ribosylation by H119; (2) polarisation of the NAD^+^ molecule for cleavage sustained by Y71 and R51; (3) stabilisation of the oxocarbenium ion resulting from NAD^+^ cleavage by M78; and (4) proton abstraction from N3 of the thymidine base by R51. The latter step represents a new mechanism of ADP-ribosylation that has not been reported for other ARTs (Figure 3C). In fact, DarT-mediated ADP-ribosylation requires the presence of both R51 and E160 residues to perform the reaction, as NAD^+^ polarisation for cleavage is promoted by R51, which, when mutated, results in a loss of DarT cytotoxicity and enzymatic activity. This mechanism is different from the canonical NAD^+^ polarisation generally found in ARTs, where it is mediated by the interaction of the 2′′ hydroxyl group of the NAM ribose with the conserved catalytic glutamate residue. These data show that a new motif also supports the ADP-ribosylation reaction, which prompts the question as to whether DarT is an early version of ARTDs or a more evolved form that specialised in ADP-ribosylation of DNA. The advance in the understanding of such peculiar DarT enzymatic catalytic mechanisms will surely help in designing specific small molecules able to modulate DarT activity. This would represent an interesting molecular target for designing future antimicrobial strategies (please see Section 4).

### 3.2. DarG Macrodomain-Containing Hydrolase Counteracts DarT Toxicity

The macrodomain-containing hydrolase DarG from *T. aquaticus* reverts DNA-ADP-ribosylation adduct on the thymidine base as observed in overexpressing *E. coli* cells with the consequent rescue of cell growth [38]. DarG antitoxins, which were characterised in *T. aquaticus* and *M. tuberculosis,* show a 56.4% sequence identity and a low sequence similarity to other bacterial macrodomain-containing hydrolases (Figure 3A). 

DarG antitoxins share a resemblance to human terminal ADP-ribose glycohydrolase 1 (TARG1), and thus belong to the phylogenetically distinct ALC1-like sub-class of macrodomains. The ALC1-like macrodomain-containing enzymes bear similarity to the macrodomain fold found in the human chromatin-remodelling enzyme, ALC1 (Amplified Liver in Cancer1), which does not possess enzymatic activity, but interacts with PAR and catalyses PARP1-dependent nucleosome remodelling upon DNA damage [120]. From a functional point of view, members of the ALC1-like class display mono-ADP-ribosylhydrolase activity by hydrolysing the acyl-ADP-ribose ester bond by lysine residue, also exemplified by TARG1. The K84 nucleophilically attacks the C1’ atom of the distal ribose, leading to the formation of a lysyl-ADP-ribose intermediate with the release of the de-ADP-ribosylated E/D residue. The lysyl-intermediate is then resolved by residue D125, which frees the ADP-ribose, and restores the K84 residue [55]. 

The DarG macrodomain adopts a typical macrodomain fold composed of a six-stranded mixed β sheet arranged between four α helices and one 3_10_-helical element. The ligand-binding pocket of the DarG macrodomain is made up of four surface loops where the bound ADP-ribose is located, and it interacts with neighbouring amino acid residues by forming hydrogen bonds (Figure 3B). The finding of W83 at the entrance of the cleft to stack with thymidine base for a correct position and K80 mostly involved in catalysis reflects the corresponding residues found in TARG1. The ligand-binding pocket is stabilised by the formation of hydrogen bonds. Key residues are found conserved, including K80, which is free to access the thymidine–ribose bond, and which is located in an equivalent position of K84 in TARG1. In fact, the mutation of K80A results in inactive DarG with a loss of hydrolase activity, similarly to the corresponding mutation on catalytic lysine in TARG1 [55]. However, the DarG catalytic mechanism remains unclear and needs further investigations. 

The great similarity in the structural fold encountered between bacterial DarG and human TARG1 (Figure 3B) suggests that TARG1 plays a role in reversing ADP-ribosylation from DNA. Experimental evidence shows that the overexpression of DarT in human TARG1 knockout cell lines induces a strong DNA damage response due to replication fork progression arrest and cell death, and that the reintroduction of TARG1 activity is required for the reversal of DarT genotoxic effects. This indicates that TARG1 is the main macrodomain enzyme in human cells that acts as a DNA repair factor analogously to DarG [121]. 

Similar reversal activity has been described for the macrodomain hydrolase SCO6735, known for its regulatory role in antibiotic production in *S. coelicolor* [102]. SCO6735 has been identified as a functional homologue of DarG as it neutralises DarT activity by hydrolysing the thymidine-linked DNA-ADP-ribosylation [119]. Structural studies have shown that SCO6735 has a notable structural similarity to *T. aquaticus* DarG and human TARG1, even though TARG1 and SCO6735 also de-MARylate protein targets while DarG does not [119]. The overall macrodomain fold in SCO6735 is conserved (Figure 3B); the superimposition of the SCO6735 crystal structure with TARG1 and DarG in complex with ADP-ribose revealed a putative active site confined by three loops. The diphosphate and distal ribose are located between two loops, namely, the phosphate-binding (PB) and substrate-binding (SB) loop. The central loop in SCO6735 is five amino acids longer than DarG and TARG1 and provides *Streptomyces* hydrolase the ability to reverse ADP-ribose from thymidine-linked ADP-ribosylation and from aspartate/glutamate-linked proteins. The catalytic mechanism relies on the correct arrangement of the V25 and Q85 residues and a catalytic water molecule within the active site that sits between these residues and the diphosphate of the ADP-ribose. The mutation of Q85, located in an equivalent position to the catalytic lysine in DarG and TARG1 (K80 and K84, respectively), leads to a complete loss of activity. These observations suggest a diversification of catalytic reaction in this sub-class of macrodomain hydrolases, providing the rationale for the design of selective inhibitors or even agonists [119].

### 3.3. DarT/DarG TA System Molecular Mechanisms and Biological Functions 

Bacterial genes encoding toxin and cognate antitoxin are frequently organised into operons, whose gene expression is regulated at a transcriptional and translational level. Under certain physiological conditions, the antitoxin protects the cell from the harmful effects of the toxin through a blockade or neutralising toxin activity [83,122,123]. Under stress conditions, the toxin is released and free to specifically impair one or more of several different cell events including DNA replication, translation, cytoskeleton formation, and membrane integrity [85,124,125]. More than 10,000 putative TA systems have been predicted by bioinformatic analyses [126,127], which can be classified into different types based on the nature of the antitoxin (non-coding RNA or protein) and on the interaction mode between the toxin and antitoxin components (Table 1) [85,125]. 

In type II TA systems, the toxin effects are mainly counteracted by the direct binding of antitoxin to cognate toxin through protein–protein interaction, forming a stable toxin–antitoxin complex [83,87,137]. As summarised in Table 2, type II toxins include endoribonucleases that target mRNA, rRNA, and tRNA; ribosome-poisoning protein acetyltransferases that target tRNAs; topoisomerase inhibitors; cell wall inhibitors; and enzymes generating PTMs that target a diverse array of cellular targets, with the majority of them involved in the downregulation of cell metabolism [138,139]. 

ADP-ribosylation is a new player in TA biology. DarT/DarG TA was initially ascribed to type II, but it is now recognised as a type II/IV hybrid system, as DarT toxicity is mainly abrogated by DarG enzymatic activity by removing the DNA-ADP-ribose adduct rather than by TA complex assembly, as detailed below. 

**Figure 4:**
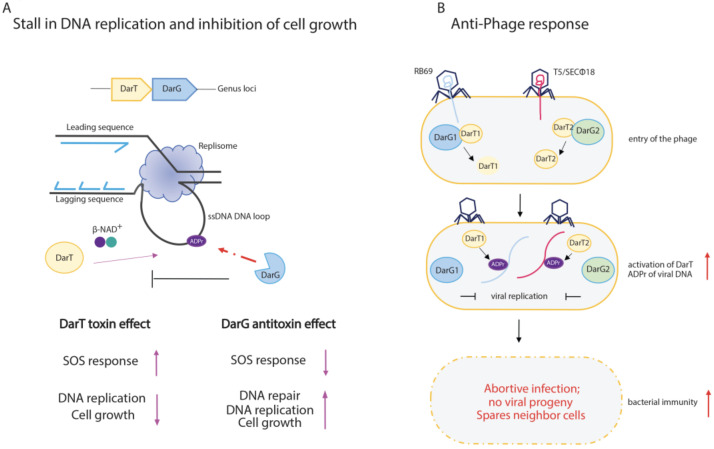
Schematic representation of DarT/DarG TA system biological functions. (**A**) DarT/DarG system in the regulation of bacterial cell growth. DarT-mediated ADP-ribosylation of ssDNA on thymidine found in consensus sequences causes a stall of DNA replication and concomitant arrest of cell growth. The activity of the DarG antitoxin counteracts DarT activity through the removal of ADP-ribose from the marked thymidine on ssDNA: DarG-mediated removal of ADP-ribose enables the replication to proceed and cell growth to re-establish. (**B**) The DarT/DarG system and the anti-phage response. Upon entry of the phages, the DarT1 and DarT2 endotoxins ADP-ribosylate viral DNA, which is unable to replicate. The overall downregulation of cell metabolic processes triggers the abortive infection programme, which leads to the host cell death and prevents viral progeny spreading in order to protect the bacterial cell population.

DarT catalyses the MARylation of NNTNTCN and TTTT/A motifs in ssDNA genomic sequences in *T. aquaticus* and *M. tuberculosis,* respectively. This enzymatic activity results in the formation of a thymidine ADP-ribose adduct that is recognised by the DNA damage repair system as a DNA lesion [38,78] (Figure 4A). It seems that *M. tuberculosis* uses this system to introduce adducts at the origin of DNA replication (OriC), which controls replication and cell growth. DarT highly ADP-ribosylates genomic DNA in DarG-depleted *M. tuberculosis* cells, leading to the activation of DNA damage response (Figure 4A). As a final outcome, the recruitment of DNAB, the replicative helicase interacting with ssDNA at the OriC and driving DNA branch migration during replication, may be impaired at cell division [38,78].

The ADP-ribosylation of genomic DNA can be counteracted by the DarG antitoxin, which reverts DNA-ADP-ribose adducts [38] (Figure 4A), thus acting as a non-canonical DNA repair factor specific for ADP-ribosyl-thymidine adducts and re-establishing bacterial cell replication. The exquisite specificity of DarG reversal has been confirmed by in vitro experiments, where human macrodomain-containing hydrolases such as MacroD1 or PARG and DraG-related ADP-ribosylhydrolase ARH3 were unable to remove ADP-ribose from genomic DNA [78]. Further investigations support the protective role of DarG against DarT-mediated toxic effects, as the activation of the DNA damage response leads to cell death in *M. tuberculosis* DarG-depleted cells [153]. The same molecular mechanisms are shared by DarT/DarG TA systems from other species, such as in the enteropathogenic *E. coli,* where the ADP-ribosylation of genomic TCT or TTT DNA sequences can affect bacterial growth and viability [40]. Notably, complementation studies show that *T. aquaticus* DarG bears mutations in the hydrolase domain, namely, in the catalytic K80 residue, yet negatively affects DarT activity, thus suggesting that the antitoxin effect of DarG can also pass through additional mechanisms [38]. Consequently, the DarT/DarG TA pair can be considered a novel hybrid TA system.

In addition to the DarG antitoxin, the DarT-mediated DNA adducts can be also repaired by the sequential action of RecF-mediated homologous recombination, which leads to the conversion of a single-strand lesion into a double-strand lesion, which is then repaired by the nucleotide excision repair (NER) pathway [40].

The finding that the DarT/DarG system is also present in other bacterial species including the pathogenic *P. aeruginosa, Acinetobacter baumannii*, and *K. pneumoniae* [40] suggests a conserved role in pathogenic bacteria. However, the triggers that specifically induce DarT toxin activity remain unknown.

### 3.4. Functional Outcomes of DarT/DarG System in Prokaryotic Immunity 

TA systems regulate several physiological processes including plasmid stabilisation and cell viability [154], persister cell formation [82,155], stress response [156], and biofilms [157] as well as multidrug resistance [86], pathogenicity [158], and defence from bacteriophages [139,159]. Overall, TA systems behave like versatile modulators of bacterial physiology that exploit the same biochemical mechanism to regulate a wide range of different activities. 

With regard to phage defence, several bacterial strains harness diverse anti-phage defence systems relying on: (*i*) the degradation of phage nucleic acids by acting through restriction endonucleases and the CRISPR-Cas system; (*ii*) abortive infection-activating mechanisms that kill the bacterial population before phage replication; and (*iii*) the inhibition of DNA and RNA synthesis through the production of small molecules with inhibitory activity, which in turn sustains bacterial immunity [160,161].

The DarT/DarG TA system modulates the anti-phage response through the ADP-ribosylation of viral DNA and the consequent induction of the abortive infection mechanism [146] (Figure 4B). As already mentioned above, abortive infection is a well-conserved mechanism within bacterial immunity, and is widespread in Gram-positive and Gram-negative bacteria, where cell death takes place prior to the maturation of the phage progeny, thus preventing the spread of phages to neighbouring cells and protecting the bacterial population. 

TA systems are known to have a pivotal role in the immunity of bacteria against phages, as their activation upon phage infection leads to cell death or growth arrest [131,162,163,164,165]. The role of ADP-ribosylation in bacterial immunity is exemplified by the DarT1/DarG1 and DarT2/DarG2 TA systems, which are found in the defence islands of the *E. coli* MG1655 bacterial genome, which are homologues of *T. aquaticus* DarT/DarG [146]. DarT toxin is conserved in both systems, whereas DarG1 and DarG2 belong to two different subfamilies; DarG1 encloses a putative YbiA-like fold, while DarG2 harbours a highly conserved macrodomain. Both DarT1/DarG1 and DarT2/DarG2 protect *E. coli* cells from natural bacteriophage infections, given that DarT1 and DarT2 are involved in ADP-ribosylation of viral DNA, with the consequent hindering of the phage’s genome replication, RNA synthesis, and assembly of mature/infective virions (Figure 4B). 

DarT1/DarG1 and DarT2/DarG2 appear to target different phages (i.e., RB69 and T5/SEC 18, respectively) and are active under different growing conditions (DarTG1 during fast growth, DarTG2 during slow growth). These findings may suggest that a different regulatory mechanism activates the two DarT/DarG systems [146]. The molecular mechanism that unleashes the DarT toxin remains elusive; yet, though a still-unknown phage-derived trigger that frees the DarT toxin to exert an anti-phage response may perhaps explain it. The finding of phage mutants that exhibit spontaneous resistance to this immunity system by interfering with DarT/DarG activity adds another layer of complexity to the bacteria–phage conflicts [146]. 

A more comprehensive understanding of DarT/DarG biology may also result in the rational design of selective phage-based therapies as an alternative to antibiotics for treating resistant pathogens by manipulating endogenous anti-phage responses [166,167]. As such, small molecules inhibiting DarT may be exploited to counteract bacterial defence mechanisms against phages, which, to date, represent the most real alternative to antibiotics.

## 4. Exploitation of DarT/DarG Biology for a Rational Design of Antimicrobial Agents

Antibiotic resistance and the recurrence of bacterial infections are two of the most urgent threats to future global public health, with implications for all areas of medicine [168]. Antibiotic treatment misuse in humans and animals has accelerated the generation of antibiotic-resistant bacterial strains. In addition, the lack of novel effective antibacterial compounds, also due to poor investment in antimicrobial research, has increased this concern [168,169]. In fact, in the past 15 years, only one new class of antibiotics against Gram-positive bacteria has been introduced into clinical practice. The majority of antibiotics on the market are based on existing drugs selected to overcome the resistance acquired by bacteria against their related compounds [170]. Therefore, in order to tackle antibiotic resistance and recurring infections, it is imperative to search for antibacterial agents that rely on innovative modes of action. 

Current antibiotics mostly target bacterial enzymes, ribosomal RNA, cell wall construction, and cell membrane function. Despite being widely used for the treatment of diverse infectious diseases, antibiotic treatments are not effective enough to eradicate highly resistant pathogens such as those referred as to ESKAPE. These resistant pathogens include *E. coli*, *S. aureus*, *K. pneumoniae*, *A. baumanii*, *P. aeruginosa*, and *Enterobacter* species, which are considered a priority by the World Health Organization for the urgent need of alternative therapeutics to antibiotic treatments. Therefore, alternative approaches to eradicate bacterial infection have been explored to deliver new therapies with clinical utility [171]. 

Mono-ADP-ribosyltransferase toxins are produced by pathogenic bacteria to infect the host cell with the impairment of vital molecular pathways [62]. These exotoxins exploit the host intracellular NAD^+^ to accomplish bacterial infection, which, in turn, causes a decrease in the level of NAD^+^ in the host, resulting in energy store depletion, immune evasion, or cell death [172]. In addition, some pathogens can also modulate NAD^+^ metabolism to support their fitness through the activity of pathogenic-specific enzymes such as NADases, or by the modulation of the activity of host NAD^+^-dependent enzymes (i.e., Sirtuins, PARPs, and CD38) [172,173]. Very recently, the pharmacological inhibition of PARPs in patients affected by diabetes mellitus has been reported to decrease intracellular *M. tuberculosis* (Mtb) in human macrophages, identifying PARP targeting as a potential novel strategy for host-directed therapy against *M. tuberculosis* and possibly against other infectious diseases [174].

With regard to NAD^+^-targeting toxins, the therapeutic inhibition of NAD^+^-dependent reactions in bacteria is still in its infancy and mainly relies on the chemical modulation of the NAD^+^ interaction pocket within the ART domain in order to block enzymatic activity. In the last two decades, antimicrobial strategies against ADP-ribosylating toxins have been proposed given that they are expected to provide new drug targets to disarm antibiotic-resistant bacteria. Different strategies, starting from the combination of PARP inhibitors, have been tested on *P. aeruginosa* Exotoxin A, *V. cholerae* Cholix toxin, *V. splendidus* Vis toxin, *S. scabies* Scabin toxin, *Bacillus cereus* Certhrax toxin, *Paenibacillus larvae* C3larvin, and Plx2A ([175] and the references therein). Such strategies have been searched for using ARTD non-specific inhibitors such as PJ34 [176], largely known for targeting human ARTDs (i.e., PARPs), polyphenolic extracts [177], and small molecules from the screening of synthetic libraries [178,179,180]. These attempts have led to the identification of lead compounds that can be further modified and explored in drug design. A promising approach relies on the use of natural compounds from plant origin that can hinder bacterial infections [181,182].

Protein–protein interactions (PPIs) have emerged as promising drug targets [183,184,185,186] and intensive efforts have led to the clinical use of PPI modulators as next-generation therapeutics in cancer treatments [187]. With regard to infectious diseases, the treatment of HIV/AIDS with the antiretroviral drugs enfuvirtide and maraviroc, which target host–pathogen interactions, provides a successful example of PPI-based drugs [188]. Since PPIs naturally occur in bacteria and regulate a multitude of cellular processes, bacterial protein–protein interactions are considered to be good candidates as a target for antibiotic drug discovery; however, to date, they are still underexplored [189,190]. 

Toxin–antitoxin systems represent a substantial pool of interactions within bacteria [83,191] that can be exploited for the development of advanced antibiotics [189,190,191]. Diverse PPI-based approaches have led to the discovery of peptides and small-molecule compounds that interfere with PPIs in TA systems, with the impairment of translation, cell wall synthesis, and lipase activity. However, no inhibitors are currently used in clinics. Given that DarG counteracts DarT activity even through the formation of a DarT–DarG complex, a PPI-based approach may be also considered in order to interfere with DarT function.

From this perspective, we have discussed the recent advances in the regulatory role exerted by the DarT/DarG hybrid TA system in the control of cell growth and abortive infection, strictly relying on ADP-ribosylation signalling. Within this framework, targeting DarT activity may represent a valuable alternative strategy for the therapeutic treatment of highly resistant pathogenic bacteria, such as *M. tuberculosis,* by preventing persistence activation. In addition, the availability of high-resolution-structure DarT provides the rationale for designing selective drugs to use as antimicrobial agents with less daunting side effects for the host. 

PARP inhibitors for the therapeutic manipulation of ADP-ribosylation have been proposed for a wide range of disorders both in human and animal models, including cardiovascular, inflammatory, autoimmune, and neurological disorders [57]. In contrast, targeting ADP-ribosylation as a therapeutic intervention to counteract infectious diseases and related antibiotic resistant bacterial strains has been much less explored, with the exception of viral-mediated disease, where ADP-ribosylation is emerging as a crucial process in host–pathogen conflicts [69,71]. The growing body of evidence for the critical role of NAD^+^ as a co-factor of enzymes involved in bacterial physiology and the pathogenic mechanism as well as in host–pathogen interactions, also including viral-mediated diseases, highlights the importance of investigating these molecular pathways in order to find novel therapeutic strategies.

## 5. Conclusions

Recent discoveries have established DNA and RNA as the novel ADP-ribosylated substrates [48,49,50]. In mammals, the reversible ADP-ribosylation of DNA is mediated by PARP1, PARP2, and PARP3, which can ADP-ribosylate phosphorylated DNA termini on ds-DNA in vitro following a phosphorylation-dependent ADP-ribosylation mechanism; however, the functional outcomes remain unknown [116,117,118,192]. 

Recently, the reversible PARP1-mediated PARylation of ssDNA that targets adenosine residues has also been identified both in vitro and in vivo, where it does not seem to be activated by DNA strand breaks [193]. Other human PARPs, such as TRPT1, PARP10, PARP11, PARP12, and PARP15, appear to target the 5’-phosphorylated end of single-stranded RNA in vitro [47,48,49,50], giving rise to a novel RNA capping mechanism. 

Several PARPs also have a role in the antiviral response through the inhibition of the virus life cycle at different stages, from transcription to translation, as exemplified by PARP7 and PARP13, which are involved in the exosome degradation of viral RNAs, and PARP12, which is responsible for the impairment of viral translation through the direct binding of viral RNA, and by the downregulation of cellular processes such as translation ([194] and the references therein). The importance of ADP-ribosylation-dependent systems in the antiviral response is highlighted by the fact that several viruses, such as alphaviruses and coronaviruses, have evolved macrodomains to counter hosts’ defensive processes controlled by ADP-ribosylation [33,53,60,195,196,197,198]. Viral macrodomains represent potential targets of antiviral drugs [69,70,71,199,200,201]. The role of ADP-ribosylation in antiviral and stress response, for instance, involving the ADP-ribosylation of viral genetic material, are reminiscent of the DNA modifications observed in lower organisms, where the transfer of ADP-ribose on nucleic acids results in the defence mechanism’s response. DarT/DarG represents one of the most ancient ADP-ribosylation-dependent systems with a role in bacterial immunity (e.g., against viral infections) as well as in the stress response. The modulation of the DarT/DarG system may also help the design of new effective antimicrobial agents in this context.

Anti-phage defence mechanisms have been extensively studied. However, many aspects still need clarification [202]. Several molecular processes underlie the anti-phage defence, which is mostly based on the degradation of the viral genome (e.g., restriction/modification enzymes, CRISPR-Cas systems, Argonaute proteins), the inhibition of DNA and RNA synthesis (e.g., chemical defence, secondary metabolite, nucleotide depletion), and abortive infection [160]. Abortive infection takes place through several molecular mechanisms, which include CRISPR-Cas and TA systems, among others [160]. A new group of retrons, belonging to prokaryotic reverse transcriptases, have been characterised to confer resistance to a wide range of phages [203,204]. Intriguingly, they share a tripartite module organisation reminiscent of TA systems, and are composed of reverse transcriptase, a multicopy single-stranded DNA (msDNA) and RcaT, an additional element protein [205]. Retrons can also be potentially used in genome editing, as they catalyse the polymerisation of DNA from an RNA template [204].

The systems mentioned above are just a few examples of the great diversity of defence systems found or predicted in bacterial cells to resist phage attack. In fact, a large number of genes encoding for different defence systems are found on the bacterial genome within chromosome regions known as “defence islands”, some of which are estimated to contain more than 100 defence genes [202]. Overall, such co-localisation of different defence genes suggests a functional link between the defence systems, including a possible coregulation mechanism. 

More than 10,000 TA systems have been found on bacterial genomes, with many bacterial species encoding dozens of TA modules. For instance, *E. coli* K12 and *M. tuberculosis* encode more than 30, and at least 80, different TA systems [146], thus suggesting that different molecular activities support TA systems in their functional outcomes. DarT/DarG represents the first TA system that induces the stress response by growth control and abortive infection by ADP-ribosylating host genomic DNA and viral DNA with the concomitant inhibition of host DNA replication and cell growth. More recently, a ParT/ParS TA system from *Sphingobium* sp. YBL2 was found to hinder nucleotide metabolism with the induction of a persistence state by ADP-ribosylating target protein [39], highlighting NAD^+^ as a key component for triggering the prokaryotic immune response [173].

The wide distribution of ADP-ribosylation systems across all domains of life highlights the importance of this modification throughout evolution [1,2,3,4,62,206,207]. Overall, we believe that our review highlights the emergence of a new and exciting research area in the ADP-ribosylation field with implications in the regulation of cellular functions still to be discovered. 

## Figures and Tables

**Table 1:** Classification of TA systems with the related targets and affected cellular functions.

TA Types	Toxin	Antitoxin	Interaction Mode	Main Targets	Affected Cellular Processes	References
Type –I	Protein	Noncoding RNA	Interference with toxin mRNA	Bacterial membrane	Biosynthesis of cell membrane	[128]
Type –II	Protein	Protein	Protein–protein interaction	DNA gyrase, EF-Tu elongation factor, genomic DNA, phosphoribosyl pyrophosphate synthetase	DNA replication, translation, nucleotide metabolism	[38,39,129,130]
Type III	Protein	Noncoding RNA	Sequestering of the toxin	mRNA	Translation	[131]
Type IV	Protein	Protein	Competition for cellular targets	Cytoskeletal proteins	Cell morphology	[132]
Type V	RNA	Protein	Hydrolysis of toxin mRNA	Cell membrane	Biosynthesis of cell membrane	[133]
Type VI	Protein	Protein	Complex formation resistant to proteolysis	β-sliding clamp	DNA replication	[134]
Type VII	Protein	Protein	Chemical modification of the toxin	Biofilm	Biofilm	[135]
Type VIII	Noncoding RNA	mRNAs	Targeting of mRNAs	YhcB inner membrane protein	Cell morphology	[136]

**Table 2:** Bacterial toxins displaying post-translational modification activity in type II TA systems.

Toxin	Bacterium	PTM Targets	Affected Biological Functions	References
HipA	*E. coli* K12	Phosphorylation of Gltx	Persistence induction	[140,141]
HipT	*E. coli* O127: H6	Phosphorylation of TrpS	Cell growth inhibition	[142]
Doc	*E. coli*	Phosphorylation of EF-Tu	Persistence induction	[130]
FicT	*P. aeruginosa*, *E. coli* and *Yersinia enterocolitica*	Adenylylation of DNA-gyrase and TopoIV	Cell growth inhibition	[143]
Fic-1	*P. fluorescens* 2P24	Adenylylation of DNA gyrase GyrB	Persistence induction	[144]
VbhT	*Bartonella schoenbuchensis*	T4SS effector	Secretion of virulence factors	[145]
DarT	*M. tuberculosis*	ADP-ribosylation of DNA	Cell growth inhibition Phage defence	[38,40,78,146]
ParT	*Sphingobium* sp. YBL2	ADP-ribosylation of Prs	Cell growth inhibition	[39]
Tre1	*Serratia proteamaculans*	ADP-ribosylation of FtsZ	Cell death	[147]
MbcT	*M. tuberculosis*	NAD^+^ degradation	Cell death	[148]
TacT	*Salmonella typhimurium*	Acetylation of tRNAs	Translation inhibition	[149]
AtaT/AtaT2	*E. coli* O157:H7	Acetylation of fMet-tRNAs	Translation inhibition	[150]
KacT	*Klebsiella pneumoniae*	Acetylation of tRNA	Translation inhibition	[151]
ItaT	*E. coli* HS	Acetylation of tRNA	Translational inhibition	[152]

## Data Availability

Not applicable.
